# Preclinical Assessment of Efficacy and Safety Analysis of CAR-T Cells (ISIKOK-19) Targeting CD19-Expressing B-Cells for the First Turkish Academic Clinical Trial with Relapsed/Refractory ALL and NHL Patients

**DOI:** 10.4274/tjh.galenos.2020.2020.0070

**Published:** 2020-11-19

**Authors:** Cihan Taştan, Derya Dilek Kançağı, Raife Dilek Turan, Bulut Yurtsever, Didem Çakırsoy, Selen Abanuz, Muhammet Yılancı, Utku Seyis, Samed Özer, Selin Mert, Cavit Kerem Kayhan, Fatma Tokat, Merve Açıkel Elmas, Selçuk Birdoğan, Serap Arbak, Koray Yalçın, Aslıhan Sezgin, Ebru Kızılkılıç, Cansu Hemşinlioğlu, Ümit İnce, Siret Ratip, Ercüment Ovalı

**Affiliations:** 1 Acıbadem Labcell Cellular Therapy Laboratory, İstanbul, Turkey; 2 Acıbadem Mehmet Ali Aydınlar University, Animal Application and Research Center, İstanbul, Turkey; 3 Boğaziçi University, Center of Life Sciences and Technologies, İstanbul, Turkey; 4 Acıbadem Maslak Hospital, Pathology Laboratory, İstanbul, Turkey; 5 Acıbadem Mehmet Ali Aydınlar University Faculty of Medicine, Department of Pathology, İstanbul, Turkey; 6 Acıbadem Mehmet Ali Aydınlar University Faculty of Medicine, Department of Histology and Embryology, İstanbul, Turkey; 7 Acıbadem Mehmet Ali Aydınlar University, Electron Microscopy Laboratory, İstanbul, Turkey; 8 Medical Park Göztepe Hospital, Pediatric Hematopoetic Stem Cell Transplantation Unit, İstanbul, Turkey; 9 Acıbadem Altunizade Hospital, İstanbul, Turkey; 10 Acıbadem Mehmet Ali Aydınlar University Faculty of Medicine, Department of Hematology, İstanbul, Turkey

**Keywords:** Chimeric antigen receptor, CD19, CAR-T, Immunotherapy

## Abstract

**Objective:**

Relapsed and refractory CD19-positive B-cell acute lymphoblastic leukemia (ALL) and non-Hodgkin lymphoma (NHL) are the focus of studies on hematological cancers. Treatment of these malignancies has undergone recent transformation with the development of new gene therapy and molecular biology techniques, which are safer and well-tolerated therapeutic approaches. The CD19 antigen is the most studied therapeutic target in these hematological cancers. This study reports the results of clinical-grade production, quality control, and in vivo efficacy processes of ISIKOK-19 cells as the first academic clinical trial of CAR-T cells targeting CD19-expressing B cells in relapsed/refractory ALL and NHL patients in Turkey.

**Materials and Methods:**

We used a lentiviral vector encoding the CD19 antigen-specific antibody head (FMC63) conjugated with the CD8-CD28-CD3ζ sequence as a chimeric antigen receptor (CAR) along with a truncated form of EGFR (EGFRt) on human T-lymphocytes (CAR-T). We preclinically assessed the efficacy and safety of the manufactured CAR-T cells, namely ISIKOK-19, from both healthy donors’ and ALL/NHL patients’ peripheral blood mononuclear cells.

**Results:**

We showed significant enhancement of CAR lentivirus transduction efficacy in T-cells using BX-795, an inhibitor of the signaling molecule TBK1/IKKƐ, in order to cut the cost of CAR-T cell production. In addition, ISIKOK-19 cells demonstrated a significantly high level of cytotoxicity specifically against a CD19+ B-lymphocyte cancer model, RAJI cells, in NOD/SCID mice.

**Conclusion:**

This is the first report of preclinical assessment of efficacy and safety analysis of CAR-T cells (ISIKOK-19) targeting CD19-expressing B cells in relapsed/refractory ALL and NHL patients in Turkey.

## Abstract (tr)

**Amaç:**

Relaps/refrakter CD19-pozitif B-hücreli akut lenfoblastik lösemi (ALL) ve Hodgkin dışı lenfoma (NHL), hematolojik kanserlerle ilgili çalışmaların odak noktasıdır. Bu malignitelerin tedavisi, daha güvenilir ve iyi tolere edilen terapötik yaklaşımlar olan, yeni gen terapisi ve moleküler biyoloji tekniklerinin geliştirilmesi ile son zamanlarda dönüşüm geçirmiştir. CD19 antijeni, bu hematolojik kanserlerde en çok çalışılan terapötik hedeftir. Bu çalışma, Türkiye’de relaps/refrakter ALL ve NHL hastalarında, ilk akademik klinik deneme için kullanılması amacıyla, CD19 eksprese eden B hücrelerini hedefleyen CAR-T (ISIKOK-19) hücrelerinin klinik sınıf üretimi, kalite kontrolü ve in vivo etkinlik süreçlerinin sonuçlarını bildirmektedir.

**Gereç ve Yöntemler:**

Bu çalışmada, insan T-lenfositleri (CAR-T) üzerinde kesilmiş EGFR (EGFRt) formu ile birlikte kimerik bir antijen reseptörü (CAR) olarak CD8-CD28-CD3ζ sekansı ile konjuge CD19 antijene özgü antikor başını (FMC63) eksprese eden bir lentiviral vektör kullandık. Hem sağlıklı donörlerin hem de ALL/NHL hastalarının periferal kan mononükleer hücrelerinden üretilen CAR-T (ISIKOK-19) hücrelerinin etkinliğini ve güvenliğini klinik öncesi olarak değerlendirdik.

**Bulgular:**

CAR-T hücre üretiminin maliyetini düşürmek için sinyal molekülü TBK1/IKKƐ’nın bir inhibitörü olan BX-795 kullanarak T-hücrelerinde CAR lentivirüs transdüksiyon etkinliğinin önemli ölçüde arttığını gösterdik. Ek olarak, ISIKOK-19 CAR-T hücrelerinin, NOD/SCID farelerinde özellikle bir CD19 + Blenfosit kanser modeli olan RAJI hücrelerine karşı önemli ölçüde ve yüksek seviyede sitotoksisite gösterdiğini değerlendirdik.

**Sonuç:**

Bu çalışma, Türkiye’de relaps/refrakter ALL ve NHL hastalarında CD19 eksprese eden B-hücrelerini hedefleyen CAR-T hücrelerinin ilk akademik klinik çalışması olarak ISIKOK-19 hücrelerinin klinik sınıf üretimi, kalite kontrolü ve in vivo etkinlik süreçlerinin sonuçlarını bildirmektedir.

## Introduction

The development of genetic modification techniques has led to the opening of a new era in cancer treatments that have been limited to conventional therapies such as chemotherapy and monoclonal antibodies for years. Host immune cells are not adequately activated by cancer cells to show a cytotoxic function because of either low anti-tumor activity or deficiency of the effector T-cells [1]. However, genetic transfer of a high-affinity chimeric antigen receptor (CAR) into autologous T (CAR-T) cells isolated from cancer patients can show effector functions without the requirement for both T-cell receptor (TCR) and major histocompatibility complex (MHC)-peptide presentation [2,3,4]. The tumor-associated antigen (TAA; for instance, CD19, BCMA, etc.)-specific CAR-T cells can recognize the TAA-expressing cancer cells and eradicate them effectively [3,4,5]. The first generation of the CAR construct was the simplest form, including an extracellular head domain from an immunoglobulin-derived single-chain variable fragment (scFv) for specific binding to the antigen of interest, which was fused to the CD3ζ signaling intracellular domain of the TCR complex [5,6]. Upon initial disappointing results with the first generation of CAR-T cells, the following generations of CARs have been improved by including one or more intracellular domain of co-stimulatory molecules such as CD28 or 4-1BB to enhance the cytotoxicity, expansion, and persistence of CAR-T cells in clinical studies [6,7,8]. The first clinical trials using transgenic CAR-T cells were performed in patients having hematological cancers including B-cell acute lymphoblastic leukemia (ALL) and non-Hodgkin lymphoma (NHL), which showed improved response rates ranging from 50% to 85% with a significant level of disease-free and overall survival [4,9,10,11,12,13,14,15,16,17,18,19,20]. Despite the efficacy of these CAR-T cells, patients could develop toxicities like cytokine release syndrome (CRS), CAR-T-related encephalopathy syndrome, B-cell aplasia, and hemophagocytic lymphohistiocytosis [14,21,22,23]. Therefore, clinical trials have been focused heavily on the safety of the therapy [21,22]. The US Food and Drug Administration, the European Union, and Canada approved Kymriah (tisagenlecleucel) by Novartis for pediatric and young adult patients with a form of ALL and Yescarta (axicabtagene ciloleucel) by Kyte-Gilead for the treatment of adults with relapsed or refractory large B-cell lymphoma for clinical use after international multi-center trials. Although the therapeutic approaches of these multi-center trials were approved, the CAR-T cell therapies are currently available only in a limited number of centers and are remarkably expensive. To overcome the limited access and the high cost of CAR-T cells, we hereby report our academic production in Turkey of CAR-T cells (ISIKOK-19) and their preclinical efficacy and safety analysis. ISIKOK-19 CAR-T cells encode the anti-CD19 CAR construct with scFv of an anti-CD19 monoclonal antibody (FMC63) conjugated with the CD8 hinge region, CD28 transmembrane (TM), and co-stimulatory domain, and the CD3ζ pro-activator signaling domain along with a truncated form of epidermal growth factor receptor (EGFRt) cell surface protein as a co-expression marker and a safety switch mechanism. In order to induce the death of CAR-T cells co-expressing EGFRt after transfusion into the body in the case of CRS side effects, cetuximab, an anti-EGFR mAb, can be administrated, which will stimulate antibody-dependent cellular cytotoxicity and complement-mediated cytotoxicity [11,24,25]. In addition, studies have reported the use of BX-795 as a lentiviral transduction-enhancing agent in several human cell lines, including NK92, Jurkat, CEM, and RPMI 8226, indicating the potential for clinical applications of gene therapy protocols [26,27,28]. This study reports the results of the clinical-grade production, quality control, and in vivo efficacy processes of the ISIKOK-19 cells as the first academic clinical trial of CAR-T cells targeting CD19-expressing B cells in relapsed/refractory ALL and NHL patients in Turkey.

## Materials and Methods

### Synthesis of CAR Construct and Lentivirus (LV) Production

The lentiviral vector (CAR-LV) encoding CD19-specific CAR (anti-CD19 scFv h(CD28-CD3ζ)-EGFRt) was designed and synthesized by Creative Biolabs. The envelope pCMV-VSV-G [a gift from Bob Weinberg (Addgene #8454; http://n2t.net/addgene:8454; RRID: Addgene_8454)] [29] plasmid, the packaging psPAX2 [a gift from Didier Trono (Addgene #12260; http://n2t.net/addgene:12260; RRID: Addgene_12260)] plasmid, and the CAR-encoding plasmid DNA were transformed into competent *E. coli* DH5α bacteria [NEB^®^ 5-alpha Competent *E. coli* (High Efficiency)]. The endotoxin-free plasmids were amplified using the QIAfilter Plasmid Giga Kit (QIAGEN), and quality control tests of the produced plasmid were performed in the Acıbadem Labmed Laboratory with accredited protocols. HEK293T cells as host cells were cultured in 5-layer cell culture flasks (NEST) for 70% confluence the day before transfection under an inverted microscope. The isolated envelope, packaging, and CAR plasmids (1:1:2 ratio) were mixed with either FuGENE HD (Promega) or polyethylenimine (PEI, Sigma Aldrich) transfection reagent for lentivirus production in Opti-MEM (Reduced Serum Media, Thermo Fisher Scientific) including 1% penicillin/streptomycin. The packaged recombinant CAR lentivirus (CAR-LV) was harvested from the supernatant of the cell cultures 48 h after transfection. The supernatant including CAR-LV was filtered (0.45 µm) and concentrated 100x with either the Lenti-X concentrator (Takara Bio) or a tangential flow filtration (TFF) system (Merck Millipore). In addition, using the TFF system, a diafiltration step was performed to reduce metabolites and small secreted proteins from the HEK293T cells based on the manufacturer’s protocol [30,31]. The CAR-LV was then prepared for transmission electron microscopy analysis. The viruses were inactivated and fixed with 2.5% glutaraldehyde in PBS (0.1 M, pH 7.2) for 2.5 h. One drop of glutaraldehyde-treated virus suspension was placed on the carbon-coated grid for 10 min. Ultrathin sections (60 nm) were stained according to the negative staining procedure. Ultrathin sections stained with 2% uranyl acetate were examined under a transmission electron microscope (Thermo Fisher Scientific - Talos L120C) and photographed at different magnification scales including 50, 100, and 200 nm.

### CAR-LV Titration and Calculation of Number of Infection Units per Milliliter (IFU/mL)

The Jurkat cell line (ATCC^®^ TIB-152™) was suspended as 10,000 cells in 100 µL of RPMI with glutamine HEPES including 10% FBS, 1% pen/strep, 1% non-essential amino acids, 1% sodium pyruvate, and 1% vitamins. Jurkat cells in 100 µL of medium were plated in 96-well plates from A to I. The wells were adjusted to have 10 µL, 3 µL, 1 µL, 0.3 µL, 0.1 µL, and 0.03 µL of the 100x-concentrated CAR-LV solutions in each 50 µL of medium, respectively, and then 50 µL of virus dilution from each concentration was transferred to Jurkat cultured wells, the total volume was adjusted to 150 µL, and cells were incubated for 3-4 days. Flow cytometry was performed using Miltenyi MACSQuant flow cytometry for EGFRt expression with anti-EGFR (cetuximab)-A488 antibody (R&D Systems) or α-Fab primary mouse antibody and α-mouse IgG-FITC secondary antibody (BioLegend). Following the CAR-LV titer assay and other quality control tests including sterility, purity, and replication-competent lentivirus (RCL) analyses, the viruses were stored at -80 °C.

### Ethics Approval and Consent to Participate

Relapsed/refractory ALL/NHL patient and healthy adult blood samples were obtained at the Acıbadem Altunizade Hospital and peripheral blood mononuclear cell (PBMC) isolation was performed at the Acıbadem Labcell Cellular Therapy GMP Laboratory within the scope of Technology and Innovation Funding Programs Directorate Grant No. 3170623. Relapsed/refractory ALL/NHL patients were recruited for the clinical study within the scope of “Study of CD19 Specific CAR Positive T-cells (CAR-T) in ALL and NHL (ISIKOK-19)” (ClinicalTrials.gov Identifier: NCT04206943). Each patient was provided with detailed information about the study and approved the patient information and consent form, which was entitled “Effectiveness of CD19 Specific CAR T-cells (ISIKOK 19) in Relapsed/Refractory ALL and NHL - Phase I/II Study.” The research was approved by the Acıbadem University and Acıbadem Health Institutions Medical Research Ethics Committee (2019-11/6).

### T Cell Transduction and Culture Conditions

CD4+/CD8+ T-cells were isolated using anti-CD4 and anti-CD8 magnetic beads (Miltenyi) following isolation of PBMCs by overlaying blood on Ficoll-Paque PLUS (GE Healthcare). T-cell activation was performed with human T-activator anti-CD3/anti-CD28 Dynabeads (Thermo Fisher Scientific) and the transduction process was initiated with the generated 100x virus titer along with Vectofusin-1 (10 µg/mL, Miltenyi MACS), protamine sulfate (40 µg/mL, Sigma), and spin down (400x g, 60 min). In addition, BX-795 (Sigma-Aldrich) was used at a final concentration of 6 µM during lentivirus transduction for 6 h to increase transduction efficiency as reported in other cell lines, including natural killer cell line NK-92 [26,27,28]. Culture and expansion processes of transduced T-cells were completed within 11-14 days in complete T cell medium [50 U/mL interleukin (IL)-2, 10 ng/mL IL-7, 10 ng/mL IL-15, 3% human AB serum, and 1% pen/strep, TexMACS Medium]. We set the CD4+ and CD8+ CAR-T cell ratio to 1:1 in the first 3 patients, while we did not adjust the ratio for the other seven patients. Total CD3+ T-cells were isolated using anti-CD3 magnetic beads (Miltenyi) for CAR-T cell manufacturing of the other seven patients. CAR-T cell production was performed in GMP units of the Acıbadem Labcell Laboratory. CAR expression level was determined by Miltenyi MACSQuant flow cytometry analysis using anti-EGFR-A488 antibody (R&D Systems).

### Quality Control Tests

Quality control tests (Tables 1 and 2) were performed based on the methods presented in the tables with the acceptance criteria [32,33]. Residual host cell protein (HCP) analysis of the CAR-LV supernatant was performed with the Cygnus Technologies-HEK 293 HCP ELISA Kit. Sterility was tested in BACTEC blood culture bottles along with the BACTEC™ FX blood culturing instrument. Endotoxin level was determined with the gel-clot endotoxin *Limulus* amebocyte lysate test (Charles River Laboratories). Impurity assay of CAR-T-cell products to determine residual pen/strep and other cell culture media components was performed with HPLC for benzylpenicillin and streptomycin at Dr. Eberhard & Partner Dortmund Laboratories in Germany. Mycoplasma analysis was performed with the *Mycoplasma *species 500 PCR kit and the GeneAmp PCR System 2700 (Applied Biosystems). Transgene CAR copy number/cell analysis was performed with the artus^®^ HI Virus-1 RG RT-PCR Kit (QIAGEN), which quantifies the HIV genome copy number per cell that accounts for genome-integrated CAR-encoding lentiviral copies in each T-cell. Efficiency of the test was confirmed with CAR-T cells and untransduced control T-cells. Quality control tests of the virus and CAR-T cells including sterility, mycoplasma, endotoxin level, transgene CAR copy number/cell analysis, and impurity assay were performed in the Acıbadem Labmed Laboratory by accredited methods. Cytogenetic analysis was performed with the standard cytogenetic banding and analyzed with the Metafer4 metasystem. Relative telomerase activity was performed with TeloTAGGG Telomerase PCR ELISA (Roche). Cytogenetics and relative telomerase activity of CAR-T cells were analyzed at Acıbadem Labgen Laboratories. Unlike the p24 ELISA assay, RCL testing was performed based on a 3-week incubation period with Jurkat cells, as confirmed before [34]. The supernatant isolated from the 3-week cultured Jurkat cells was then reincubated with untransduced fresh Jurkat cells and the CAR expression level was analyzed by flow cytometry. The acceptance criterion for this assay was to detect ≤1% CAR expression. CAR-T cell stability analysis was performed by cell viability determination following 24 h of incubation.

### In Vitro Anti-Tumor Cytotoxicity and Efficacy Assay

Preclinical in vitro studies with CAR-T cells were performed for the assessment of efficacy and cytotoxic capacity. For that purpose, anti-CD19-expressing CAR-T cells and CD19-expressing RAJI cells (Burkitt’s lymphoma, CCL-86, ATCC^®^) were co-cultured for 24 h, 7 days, and 14 days (effector:target; 1:1, 5:1, 10:1) prior to labeling target cells including RAJI, DAUDI (ATCC^®^ CCL-213™), and K562 (ATCC^®^ CCL-243) cell lines with either the CellTrace™ Violet Cell Proliferation Kit (Thermo Fisher Scientific) according to the manufacturer’s instructions or α-CD19-PE (BioLegend) and α-CD19-PE.cy7 (Miltenyi) for the analysis with flow cytometry. At the end of the co-culture process, α-EGFR-FITC (Abcam) and 7-AAD (Invitrogen) were evaluated for cytotoxicity. CAR-T cell activation was determined with the upregulation of CD25 (IL2RA, IL-2 receptor alpha chain) and CD107a (marker for degranulation of lymphocytes) on CAR-T cells using α-CD3-PE, α-CD4-Viogreen, α-CD8-Vioblue, α-CD25-APC, and α-CD107a-PE.cy5.5 (Miltenyi) with MACSQuant flow cytometry (Miltenyi). After 24 h of co-culturing, supernatants from cultured cell conditions were also collected and IFNγ cytokine levels were assessed with the Human IFNγ ELISA Kit (Thermo Fisher Scientific) according to the manufacturer’s instructions. The ELISA plate was measured at 450 nm and 550 nm using a microplate reader (BMG LABTECH).

### Anti-Tumor Efficacy and Safety in In Vivo Cancer Model of NOD/SCID Mice

Cells of a CD19-expressing Burkitt’s B-cell lymphoma line, RAJI [13], were transduced with fLuciferase-mCherry-encoding lentivirus, which enabled the sorting out of  >98% mCherry-positive RAJI cells using flow cytometry. This experimental model consisted of four groups and each group had 5 NOD/SCID mice (male or female, 6-8 weeks old), including tumor-only, control T, single-dose CAR-T (1x10^6^ CAR+T), and double-dose CAR-T groups. The 5x10^5^ fLuciferase-mCherry-positive RAJI cells were then injected intraperitoneally (IP) into each NOD/SCID mouse in all groups at day 0. Bioluminescent positive tumors were determined within 3 days after the injection. At day 3, 5x10^6^ control T-cells or an equal amount of a mixed T cell population containing 1x10^6^ CAR-T cells and 4x10^6^ control T-cells (equal to 20% CAR expression in the population), and on the 10^th^ day for the double-dose CAR-T cell group a further CAR-T cell dose (5x10^6^ of the mixed T cell population including 1x10^6^ CAR-T cells), were injected into each mouse (or only normal saline solution in the tumor-only group). Tumor growth and development were monitored by bioluminescence with Luciferin (1 mg/mL) injection. The emitting bioluminescence signal of tumors was measured with the IVIS in vivo imaging system (Caliper Lumina 3, Boğaziçi University Life Sciences Center). All groups were monitored until day 49. Following this period, the mice were only screened for survival rate until day 60. All mice experiment methods followed the protocols approved (ACU-HADYEK 2017/31) by the Acıbadem Mehmet Ali Aydınlar University Laboratory Animal Application and Research Center (ACUDEHAM), where they were performed.

### Histopathological Analysis

Dissected organs including the intestines and peritonea of sacrificed mice were placed into 10% buffered formalin solution prior to being sent for routine tissue processing. Each organ was taken into a cassette to create a paraffin block.  The blocks were cut into a thickness of 4 µm and stained with hematoxylin & eosin (H&E). H&E slides were analyzed for the absence/presence of tumors initially, and then the percentage of tumor burden was determined for each organ. A second analysis was performed in order to determine absence/presence of necrosis in each organ.

### Statistics Analysis

Two-tailed Mann-Whitney U tests were performed using GraphPad Prism software for bar graphs. Outliers were not excluded in any of the statistical tests and each data point represents an independent measurement. Bar plots report the mean and standard deviation or the standard deviation of the mean. The threshold of significance for all tests was set at p<0.05. The survival probability of all experimental groups (5 mice per group) was calculated using Kaplan-Meier methods throughout the 60-day period as previously described [35,36,37].

## Results

### Construction of Anti-CD19 CAR Gene and Process Optimization of Lentivirus Production

**Figure 1:**
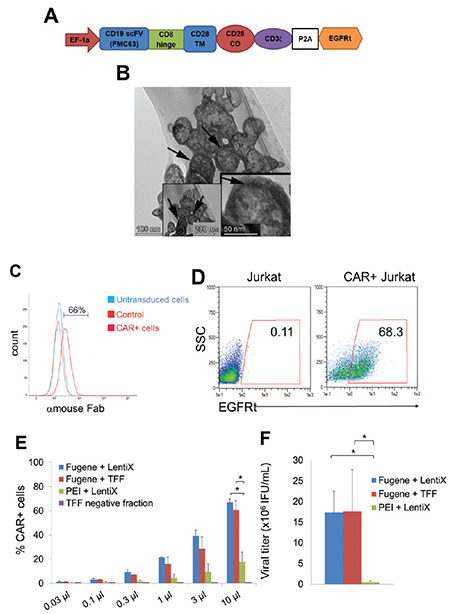
Construction of the anti-CD19 CAR-EGFRt gene and process optimization of CAR-LV production. A) The αCD19-CAR construct in the pCDCAR1 lentiviral vector was transcribed under the EF-1α promoter, which consists of the αCD19 scFv (FMC63 clone) head domain conjugated with CD8 hinge, CD28 transmembrane (TM), and co-stimulatory domains (CD), and the CD3ζ pro-activator signaling domain along with the P2A auto-cleavage peptide and a truncated form of EGFR (EGFRt) cell surface domain as a co-expression marker. B) Representative electron micrographs of lentiviruses showing a group of virus particles (arrow) in the sections (scale bars: 200 nm, 100 nm, 50 nm). C) The flow histogram showing the CAR-expressing control and untransduced cells that were stained with the Fab region of the CAR-specific antibody as a two-step antibody staining protocol. D) Flow cytometer plots showing Jurkat cells that were either untransduced or transduced with αCD19-CAR-EGFRt that were probed with the αEGFR-A488 antibody. E) Plot showing the CAR expression of Jurkat cells transduced with CAR-LV in a dose-dependent manner at day 4 of transduction. Viral particles were produced with FuGENE or PEI transfection reagent in HEK293T. The viral titer was concentrated to 100x using the Lenti-X concentrator reagent or TFF device. F) Bar graph showing titers of the CAR-LV virus that were produced with FuGENE + Lenti-X concentrator (blue bar), FuGENE + TFF concentration system (red bar), or PEI + Lenti-X concentrator (green bar) as x106 IFU/mL. Means and standard deviation ranges of at least three independent experiments are shown. *: p<0.05.

A CD19 antigen-recognizing CAR synthetic gene construct was established in the second generation of the lentiviral vector by Creative Biolabs as a CD19 antigen-specific antibody head (clone FMC63) along with a CD8 hinge, CD28 TM, and co-stimulatory domains (CD), and the CD3ζ pro-activator signaling domain (Figure 1A). The CAR construct was also conjugated with the P2A auto-cleavage peptide and the extracellular domain of the *EGFR* gene as a truncated form (EGFRt) to assess CAR expression and also as a “safety switch” in the case of severe CRS [24,38] (Figure 1A). The CAR-encoding lentiviral vector was transfected to the HEK293T cell line in multi-layer flasks as described in the Materials and Methods section for production of the α-CD19-CAR-EGFRt lentivirus (CAR-LV). The majority of the cellular impurities in the lentiviral products were removed by TFF concentration and diafiltration process, and their safety and efficacy (Table 1) were confirmed. However, empty and non-therapeutic particles that lack CAR-encoding vector RNA are other significant impurities that can cause viral component-related toxicity and immunogenicity compromising safety and efficacy [39]. Transmission electron microscopy analysis could confirm the formation of complete/therapeutic and empty/non-therapeutic particles [39]. In order to determine complete particles of the CAR-LV in the final product, which was purified using TFF, frozen at -80 °C, and thawed, representative electron micrographs of CAR-encoding lentiviruses were taken. They showed high amounts of complete particles that were captured as a group of concentrated virus particles in the sections (Figure 1B). Afterwards, CAR expression in cells was determined upon transduction using the CAR Fab-specific antibody and secondary antibody (Figure 1C). CAR expression on cells was assessed using EGFRt-specific antibody Figure 1D), as other methods such as Fab antibody staining and analysis of direct CAR are time-consuming and the low binding capacity of the antibody made the proper visualization of CAR-expressing cells difficult (Figure 1C). As CAR-expressing T and untransduced T-cells could be distinguished by using one-step staining with the α-EGFR, the same staining procedure was employed throughout the study. In order to optimize the production process, concentration, and efficacy of the CAR-LV, different transfection reagents or concentration procedures including FuGENE or PEI transfection reagents along with either the Lenti-X concentrator solution or TFF system were tested. In Figure 1E, the testing of CAR expression on Jurkat cells, which were transduced dose-dependently with CAR-LV that had been produced and concentrated in different ways, is shown. As a first step, the FuGENE transfection reagent (17.4x10^6^±5.1x10^6^ virus titer) was determined to be significantly more efficient than PEI (0.44x10^6^±0.31x10^6^ virus titer) (Figures 1E and 1F). Also, both Lenti-X and TFF were shown to concentrate the 100x CAR-LV at a very high recovery rate (>95%) in comparison to the TFF negative fraction whether by assessing transduction efficiency (Figure 1E) or viral titer (Figure 1F). However, TFF can also perform diafiltration to remove metabolites and small secreted proteins from HEK293T cell supernatant as well as the residual Benzonase, which was used as a nuclease treatment to degrade plasmids and other nucleic acid contaminants [30,31,40]. Therefore, for efficient and clinical-grade CAR-LV production according to the acceptance criteria [32] (Table 1), the FuGENE transfection method was utilized, followed by a TFF concentration and diafiltration step.

### In Vitro Activation and Cytotoxicity Assay of the Produced CAR-T Cells

**Figure 2:**
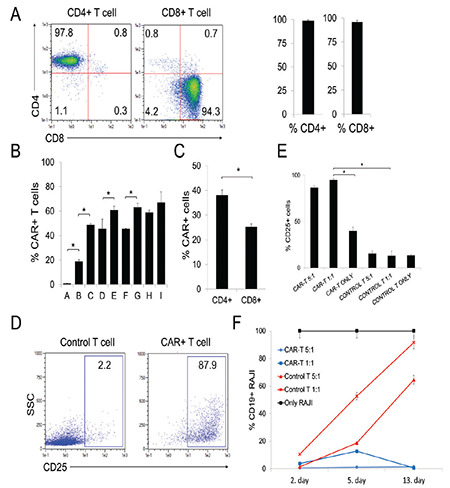
In vitro activation and cytotoxicity assay of the produced CAR-T cells. A) Flow cytometer plots showing CD4+ or CD8+ T cells isolated from 3 healthy donors. Bar graphs show CD4 or CD8 expression in T cells after sorting. B) Quantification of CAR expression on T cells that were from the untreated control (A bar) or transduced with 2-MOI CAR-LV (B bar), LV + spinoculation (Spin) (C bar), LV + protamine sulfate (Ps) (D bar), LV + Ps + Spin (E bar), LV + Vectofusin (Vec) (F bar), LV + Vec + Spin (G bar), LV + Vec + Ps (H bar), or LV + Vec + Ps + Spin (I bar). *: p<0.05. C) Quantification of CAR expression either on CD4+ or CD8+ T cells from 3 healthy donors with 1-MOI LV at day 12. *p<0.05. D) Flow cytometer plots show the upregulation of CD25 activation marker on CAR-T cells compared to conventional control T cells co-cultured with RAJI cells for 48 h. E) Quantifications of the CD25 upregulation on either CAR-T or control T cells co-cultured with RAJI at either 1:1 or 5:1 ratio or without the target cells for 48 h. *: p<0.05. F) Quantifications of viable RAJI cells upon culturing with either CAR-T or control T cells at day 2, 5, or 13. Means, standard deviation range, and the data of at least three independent experiments with PBMCs from healthy adult donors are shown.

Following the CAR-LV production, PBMCs were isolated from healthy human donors. Following this, CD8+ (95.9±1.4%) and CD4+ (98.9±0.99%) T-cells were positively selected using α-CD8 and α-CD4 magnetic beads (Figure 2A). Afterwards, both CD8+ and CD4+ T-cells were activated using α-CD3/α-CD28 Dynabeads for 24 h before transduction with CAR-LV. As a first step, activated T-cells were either untreated as the control or were transduced with CAR-LV along with combinations of different treatments including spinoculation, protamine sulfate, and Vectofusin-1 (Figure 2B). Figure 2B shows that spinoculation (48.7±1.1%; C bar), protamine sulfate (45.7±7.7%; D bar), and Vectofusin-1 (45.7±0.4%; F bar) alone were able to increase the CAR expression by more than ~2.5-fold with respect to only CAR-LV transduction (18.9±1.5%; B bar). On the other hand, combinations of  treatments such as protamine sulfate and spinoculation (61±3.1%; E bar), Vectofusin-1 and spinoculation (63±3.2%; G bar), and protamine sulfate and Vectofusin-1 (58.9±2%; H bar) significantly increased the CAR expression by ~3.4-fold with respect to only CAR-LV (Figure 2B, B bar). The highest CAR expression (67.2±8.6%; I bar) with CAR-LV multiplicity of infection (MOI) of 2 was achieved when spinoculation, protamine sulfate, and Vectofusin-1 were all applied together, although this was statistically insignificant compared to other combinations of the manipulations (E, G, and H bars) (Figure 2B). In subsequent CAR-T cell productions, activated CD4+ and CD8+ T-cells from 3 healthy human donors were transduced with CAR-LV of 1 MOI along with spinoculation, protamine sulfate, and Vectofusin-1 treatments. Upon 20 h of incubation with the virus suspension, cells were washed with PBS and cultured in the G-rex 100M platform for 14 days for gas-permeable rapid expansion. The cells were cultured along with a complete T cell medium including IL-2, IL-7, and IL-15 cytokines, which were reported to promote the differentiation, persistence, and expansion of stem-cell memory T (T_scm_) and central memory T (T_cm_) cells [41]. The CAR expression in CD4+ T-cells was 38.1±2%, while 25.2±1.3% CAR expression was recorded for CD8+ T-cells (Figure 2C). Furthermore, to test effector functions including activation and cytotoxicity of the CAR-T cells, CD4+ and CD8+ CAR-expressing T-cells were combined in a 1:1 ratio. Subsequently, CD19 antigen-expressing RAJI cancer cells [9] were cultured with the CAR-T cells in different effector:target (E:T) ratios of 1:1 and 5:1 for 13 days. Specific activation of CAR-expressing T-cells was determined with the upregulation of CD25, which is an activated proliferating lymphocyte marker [42], in comparison with the untreated control T-cells in the same culture (Figure 2D). CD25 upregulation in CAR+ T-cells was shown to be significantly high (88.9±1.7%) at the 5:1 ratio and was 96.4±1.5% at the 1:1 ratio in comparison with the control T-cells cultured at a 1:1 ratio (15.3±4.9%) (Figure 2E). At the 13^th^ day, the viable CD19+ RAJI population was shown to have increased by more than 90% and 60% in the co-culture with control T-cells at 1:1 and 5:1 ratios, respectively (Figure 2F). However, the viable RAJI cells were less than 1% at the 13^th^ day in co-culture with CAR-T cells at both E:T ratios (Figure 2F). CAR-T cells were found to exhibit the best cytotoxic activity at the high CAR-T dose (5:1) in the co-culture tests with RAJI cells (Figure 2F). These efficacy tests showed that the studied CAR-T cells (ISIKOK-19) were specifically activated and highly cytotoxic against CD19 antigen-expressing RAJI cells. A full list of the quality control tests accepted internationally in the relevant literature [32] showed that all three lots were found to comply with the acceptance criteria (Table 2). In order to assess the clinical-grade quality of the CAR-T cells, sterility tests (including microbial growth, mycoplasma, and endotoxin), tumorigenicity tests (including relative telomerase activity and cytogenetic analysis), RCL testing, and impurity analysis (including residual α-CD3/α-CD28 beads and residual penicillin-streptomycin) were performed and results were determined to comply with the acceptance criteria (Table 2). Following this, in vivo efficacy analysis of the CAR-T cells in NOD/SCID mice was planned using the fLuciferase-mCherry-expressing RAJI cancer model.

### In Vivo Efficacy of CAR-T Cells in NOD/SCID Mice with RAJI Cancer Model

**Figure 3:**
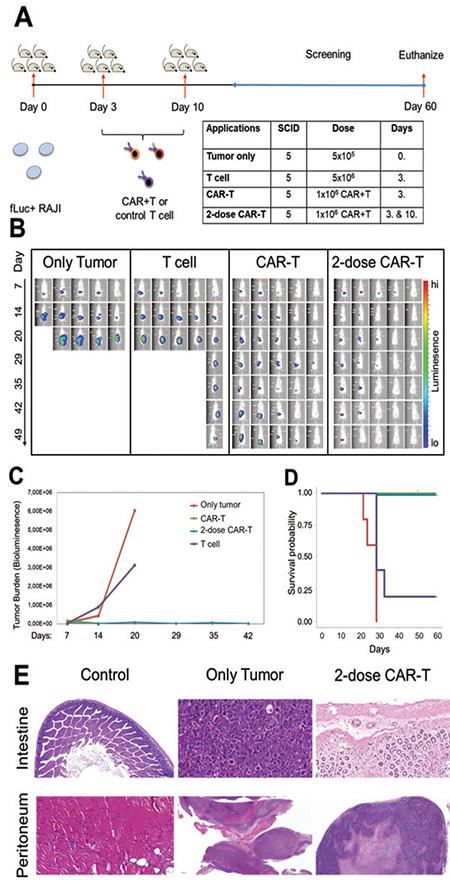
In vivo efficacy of CAR-T cells in NOD/SCID mice with RAJI cancer model. A) Experimental plan whereby NOD/SCID mice were injected with fLuciferase-expressing RAJI cells at day 0 and beyond, with normal saline to the “tumor-only” group and single-dose CAR-T cells, double-dose CAR-T cells, or control T cells following RAJI cell administration. All groups were screened with bioluminescence until day 49 and animals were euthanized on day 60. B) Bioluminescence radiance of all mice in the groups. C) Quantification of the tumor burden in the groups (tumor-only group, red line; T cell infusion group, purple line; single-dose CAR-T group, green line; double-dose CAR-T group, blue line) that were assessed by level of bioluminescence for 42 days. D) Mean of survival probability rates of the groups based on screening for 60 days. E) Histopathology images of intestinal and peritoneal tissues of the mice from the untreated control group, tumor-only group, and double-dose CAR-T cell group.

Preclinically efficient CAR-T cells were next tested in an in vivo cancer model with NOD/SCID mice. Figure 3A shows the in vivo experimental approach, where fLuciferase-mCherry-positive RAJI cells, control T-cells, single-dose CAR-T cells, and double-dose CAR-T cells were transferred to NOD/SCID mice. The growth rates of the fLuciferase-expressing RAJI cells in the mice were tracked using luciferin for in vivo bioluminescence screening for 49 days (Figure 3B). The first death was observed in the in vivo study in the tumor-only group on day 20 (Figure 3B). At the end of the first month, all mice in the tumor-only group and 4 of 5 mice in the group administrated T-cells were dead (Figure 3B). However, tumor burden as measured by the level of bioluminescence was decreased significantly (>90%) in both the single-dose and double-dose CAR-T cell groups (single-dose group: 6.7%, double-dose group: 1.3%) in comparison with the tumor-only group (Figure 3C). The tumor burden level was decreased 15-fold with the single-dose CAR-T cell transfer and 75-fold with the double-dose CAR-T cell transfer in comparison with the tumor-only group (Figure 3C). The tumor load in the control T cell-administrated group was also significantly higher (50%) than that in both CAR-T cell groups (Figure 3C). The mean survival was 24 days in the tumor-only group and 27 days in the group that received T-cells (FigureS 3B and 3D). No deaths were observed in either the single-dose or double-dose CAR-T treatment groups as the survival was screened for 60 days until the mice were euthanized (FigureS 3B and 3D). In the in vivo efficacy experiment, both single- and double-dose infusions of the CAR-T cells to the mice groups significantly increased the survival rate and decreased tumor growth in comparison with the tumor-only group and the control T cell-treated group. Furthermore, an increment in the activity of the CAR-T cells in a dose-dependent manner was shown with the double-dose of CAR-T cell therapy. At the end of the 60-day screening for survival rate, the tumor burden and toxic effects of CAR-T cells in the mice were assessed. In order to determine the CAR-T cell toxicity in the groups, histopathology analysis was done in a subsequent experiment. In the intestine and peritoneum, tumor formation and necrosis were clearly demonstrated, both in the tumor-only group and the T cell control group, but not in the untreated control group and double-dose CAR-T group (Figure 3E). This suggests that the anti-tumor effects of the CAR-T cell administrations were consistent with the in vivo bioluminescent screening of the tumor burden. Furthermore, double-dose CAR-T cell infusion and its cytotoxicity against the cancerous cells did not cause necrosis in the tissues of the mice (Figure 3E).

### Efficacy and Cytotoxicity Capacity of Patient Blood-Derived CAR-T Cells

**Figure 4:**
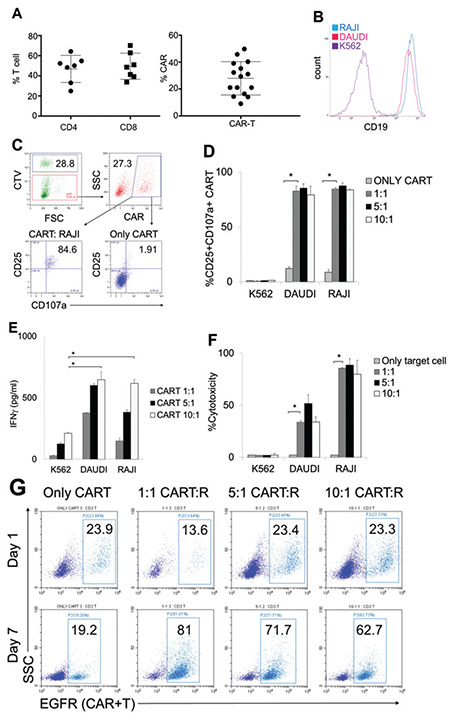
Efficacy and cytotoxicity capacity of the CAR-T cells produced from ALL/NHL patients’ PBMCs. A) Bar graph (left) showing proportions of CD4+ and CD8+ T cells following CD3+ T cell isolation from PBMCs of seven patients. Bar graph (right) showing CAR expression in the T cells isolated from blood of 10 patients. The lines across the bars show the lowest and highest CAR expression achieved during CAR-T cell production. B) Histogram of CD19 expression in cell lines including RAJI, DAUDI, and K562, which were later used in cytotoxicity experiments as target cells. C) Flow cytometer plots of the CAR-T cells and the CTV-labeled target cells cultured at 1:1, 5:1, and 10:1 ratios (effector:target cell) for 24 h. Activation of the CAR-T cells was determined in flow cytometer analysis with the upregulation of CD25 and CD107a. D) Activation level (CD25+CD107a+) of the CAR-T cells that were cultured for 24 h either with the target cells at different ratios or without the target cells. E) IFNγ secretion capacity of the CAR-T cells upon co-culturing (dashed bar, only CAR-T; light gray bar, 1:1; black bar, 5:1; dotted bar, 10:1) for one day. F) Cytotoxicity capacity of the CAR-T cells against the target cells after 24 h of co-culturing. G) CAR (EGFR+) expression flow cytometer charts of the CAR-T cells cultured with or without RAJI cells for up to 7 days. Means, standard deviation ranges, and the data of at least three independent experiments with PBMCs from different ALL/NHL patients are shown.

It is important to demonstrate that the cytotoxicity potential of the ALL/NHL patient-derived CAR-T cells is not inferior to that of healthy adult blood-derived CAR-T cells. PBMCs from 10 patients with relapsed/refractory ALL/NHL were isolated in order to investigate this. We set the CD4+ and CD8+ CAR-T cell ratio to 1:1 in the first 3 patients, while we did not adjust the ratio for the other seven patients. We abandoned the method of mixing the CD4+ and CD8+ CAR-T cells because of the costs of producing CD4+ and CD8+ CAR-T cells separately. Proportions of CD4+ T cells were 46.8±13.4% and those of CD8+ T-cells were 49.5±13.1% for the CD3+ T-cells isolated from the patients’ PBMCs (Figure 4A). CAR-T cells (at 4 to 6x10^6^ CAR-T cells per kilogram of patient weights) were manufactured from the isolated T-cells at 9-12 days. CAR expressions from the patient-derived T-cells ranged between 10% and 50% (Figure 4A). Four out of 15 manufactured CAR-T cells showed <20% CAR expression, lower than specified by the acceptance criteria (Figure 4A). Next, we increased the CAR-LV doses to 3 MOI rather than 1-2 MOI during transduction for subsequent CAR-T cell productions, which achieved 30%-50% CAR expression (Figure 4A). We successfully manufactured CAR-T cells with a yield of 4 to 6x10^6^ CAR-T cells per kilogram for all ten patients from their isolated CD3+ T-cells at 9-12 days. Subsequently, in order to test activation potentials and cytotoxicity capacities of the CAR-T cells, the cells were co-cultured with CD19-positive (RAJI and DAUDI) or CD19-negative (K562) target cells (Figure 4B). Activation of CAR-T cells was determined in the presence of the CTV-labeled target cells with upregulation of CD25 and CD107a co-expression (Figure 4C), which are increased on the surface of activated and cytotoxic lymphocytes [42,43,44]. CAR-T cells co-cultured with CD19-expressing DAUDI and RAJI cell lines had highly upregulated CD25 and CD107a, except for the CAR-T cells co-cultured with the CD19-negative K562 cell line (Figure 4D). IFNγ cytokine secretion from the activated CAR-T cells [12,22,43,45] was also investigated and results showed that the IFNγ secretion level was increased with higher doses of CAR-T cells (5:1 and 10:1 ratios co-cultured with RAJI or DAUDI) in comparison with the cells co-cultured with K562 target cells (Figure 4E). On the other hand, we determined some non-specific IFNγ secretion in the culture conditions with K562 in a dose-dependent manner for CAR-T cells (Figure 4E). We conclude that this unspecific IFNγ secretion came from the total T-cells activated with α-CD3 and α-CD28 beads for the expansion of CAR-T cells for 12 days, which might lead to some unspecific secretion. These data showed that CAR-T cell activation specifically depends on CD19 expression on the target cells. Furthermore, cytotoxicity of the activated CAR-T cells was determined against DAUDI and RAJI cells, but not for K562 cells, further confirming the specificity to the presence of CD19 antigen (Figure 4F). Frequency of CAR-expressing T-cells was subsequently assessed by culturing CAR-T cells with RAJI cells at different doses (1:1, 5:1, 10:1, or only CAR-T cells without RAJI cells) for up to 7 days. Activated CAR-T cell proportion in total CD3+ T cells was found to increase 2- to 4-fold when compared to 7-day culturing with target RAJI cells at all different ratios, but not in T-cells cultured without RAJI cells (Figure 4G). This finding suggested that activated CAR-T cells specifically proliferate in the T-cell population in comparison with the untransduced T-cells. In conclusion, patient-derived CAR-T cells possess high CD19-specific activation and cytotoxicity potential along with proliferative capacity.

### Upregulation of CAR Transduction Efficiency with BX-795 in CAR-T Cell Production

**Figure 5:**
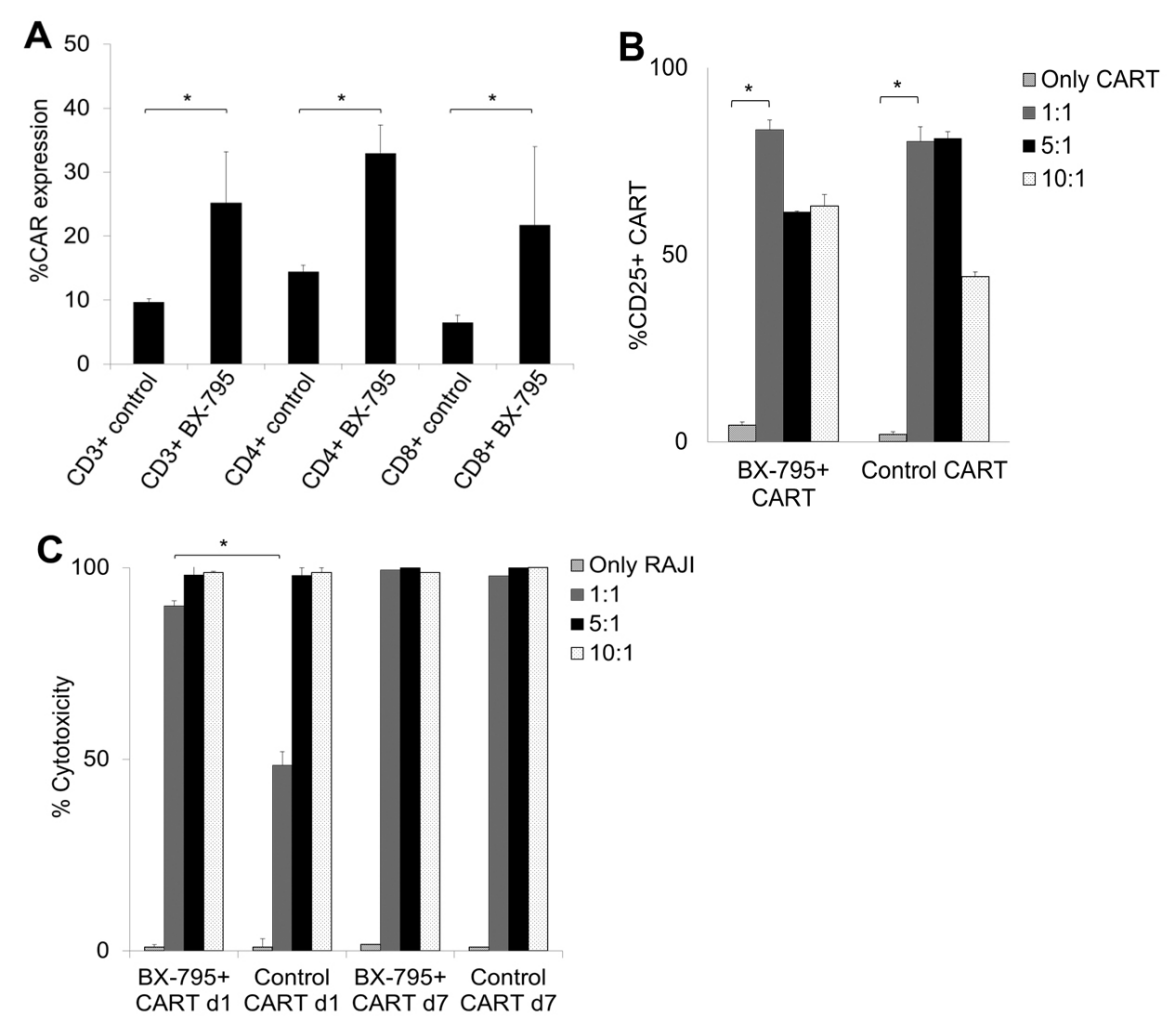
BX-795-dependent increase in CAR-LV transduction efficiency in T cells. A) Bar graph of CAR expression in total CD3+ T cells, CD4+ T cells, and CD8+ T cells assessed at day 5 upon 1-MOI CAR-LV transduction with or without 6 μM BX-795. B) Upregulation of activation marker CD25 in CAR-T cells (produced with or without BX-795) that were co-cultured with RAJI cells at different effector:target ratios for 24 h. C) Bar graph showing the cytotoxicity levels of RAJI cells co-cultured with CAR-T cells (produced with or without BX-795) for 1 to 7 days. Means and standard deviation ranges of at least three independent experiments with PBMCs from different relapsed/refractory ALL/NHL patients are shown.

Lower amounts of virus can be used to produce the same numbers of CAR-T cells by increasing CAR transduction to T-cells with low MOI of lentivirus. This would reduce the cost of CAR-T cell manufacturing and provide a more cost-effective T-cell gene modification process [46]. In order to achieve efficient CAR-T cell production, BX-795, an inhibitor of the TBK1/IKKe complex acting downstream of RIG-I, MDA-5, and TLR3, which was previously shown to increase the transduction efficiency of lentivirus to human cell lines including the Jurkat T-cell line and NK92 natural killer cell line [26,27,28], was tested.  α-CD3/α-CD28-stimulated CD3+ T-cells isolated from ALL/NHL patients’ blood were expanded for 10-12 days with CAR-LV of 1 MOI and 6 µM BX-795 for 6 h before washing twice with complete CAR-T media. CAR expression was determined to be significantly increased (~2 fold) in not only the total CD3+ T-cell population but also the CD4+ and CD8+ T-cell populations in comparison with T-cells transduced with 1-MOI CAR-LV in the absence of BX-795 (Figure 5A). The next question to evaluate was whether there was a difference in the activation capacity and cytotoxicity potential of the CAR-T cells produced with and without BX-795. The study showed that CAR-T cells produced in the presence of BX-795 (BX-795+ CAR-T) could be significantly activated with RAJI cells at levels as high as those of the control CAR-T cells at all E:T ratios (Figure 5B). Subsequently, cytotoxicity of both BX-795+ and control CAR-T cells against RAJI cells after up to 7 days of co-culturing was assessed. BX-795+ CAR-T cells were found to have significant cytotoxicity as high as the control CAR-T cells and both of them could kill the target cells up to 98% at the end of 7 days of co-culturing (Figure 5C). Furthermore, at 1:1 CAR-T to RAJI doses, BX-795+ CAR-T cells showed significantly higher cytotoxicity than control CAR-T cells (Figure 5C). These data suggest that CAR-T production using BX-795 did not affect activation efficacy and cytotoxicity capacity of the CAR-T cells.

## Discussion

Genetically engineered T-cells that express TAA-specific CAR have shown significantly high response rates for cancers including B-cell ALL, NHL, chronic lymphocytic leukemia, and multiple myeloma with impressive overall survival rates [4,9,10,11,12,13,14,15,16,17,18,19,20,47]. CD19-expressing hematological malignancies are the most studied cancers with antigen-specific immunotherapy, as CD19 expression is restricted to B cells and follicular dendritic cells [48,49]. Immunotherapy trials with CD19-antigen binding scFv conjugated CAR-T cells (α-CD19 CAR-T) have shown high levels of cytotoxic function against CD19-positive B cell malignancies [20,50]. This study reports the in vitro and in vivo experimental efficacy of our produced clinical-grade α-CD19 CAR-T cells, namely ISIKOK-19. Our findings prompted us to launch the first academic phase of CAR-T cell clinical trials with ALL and NHL patients in Turkey.

For large-scale lentiviral production, adherent HEK293T cells were transfected in multi-layer T175 flasks along with either PEI or FuGENE transfection reagents. Although the PEI transfection method is highly cost-effective, the FuGENE transfection method achieved 50- to 100-fold increases in virus titers. This allowed production of 1 dose of CAR lentivirus per patient in a time-efficient manner. Although this optimization of virus production is thought to be suitable for an academic clinical trial, other systems, including the use of bioreactors along with suspension HEK293T systems, can increase CAR-T cell production to allow treatment of higher numbers of patients.

Optimization of CAR-T cell production was also planned in this study. In order to increase the efficiency of CAR lentivirus transduction, protamine sulfate and Vectofusin-1 along with the spinoculation method were comparatively tested regarding lentivirus transduction of T-cells. Findings of this study showed that binary combinations of these three treatments significantly increased CAR transduction. This will allow the use of smaller amounts of virus for future CAR-T cell production. Therefore, large-scale lentivirus and CAR-T cell production optimizations will lead to quicker and easier processes and lower costs.

Various α-CD19 CAR constructs have been tested to determine the specificity, effector function capacity, and persistence of the CAR-T cells in preclinical as well as clinical studies [8]. The FMC63-28-ζ CAR construct has been associated with high levels of CAR expression and cytokine secretion upon CD19-antigen recognition [5]. The findings of this study showed that a high dose (5:1, effector:target) of CAR-T cells in culture with RAJI cells resulted in CAR-T cell persistence and control of tumor growth in in vitro experiments in 2 weeks. Similarly, double-dose CAR-T cell treatment achieved the longest tumor-free state with undetectable levels of bioluminescence for at least 7 weeks in 4 out of 5 mice in comparison with the single-dose CAR-T treatment. This suggests a significantly long persistence of the CAR-T cells in vivo. Nonetheless, persistence of CD3+ cells in the blood in the mouse model was not determined in this study. Among clinical trials, splitting up the total dose of CAR-T cells (for BCMA) over multiple injections (for instance, three injections at 10%-30%-60% of the total, each one day apart) reduced severe events like CRS or neurotoxicity and increased the persistence of treatment [25,51,52,53]. We also manufactured CAR-T cells with patient-derived T-cells within the scope of the clinical trial “Study of CD19 Specific CAR Positive T-cells (CAR-T) in ALL and NHL (ISIKOK-19)” (ClinicalTrials.gov Identifier: NCT04206943). Findings of that study showed that ISIKOK-19 CAR-T cells manufactured from 10 relapsed/refractory ALL/NHL patients’ blood managed to eliminate CD19-positive RAJI and DAUDI B cell target models efficiently and specifically, with the exception of the CD19-negative K562 cell line. We showed that 4 out of 15 manufactured CAR-T-cells had 10%-20% CAR expression. In our first productions, we used CAR-LV of 1-2 MOI, which might not be enough. As the literature reports [54], this difference in CAR expression might be caused by the viability, activation, and proliferation capacities of the T-cells isolated from different donors and from the lentivirus lots produced individually for each patient. Patient-derived CAR-T cells were manufactured in a reproducible manner, at amount of 4 to 6x10^6^ CAR-T cells per kilogram, which is the amount utilized for treatments in clinical trials [55]. Furthermore, similar to previous studies [26,27,28], using the inhibitor drug BX-795, the transduction efficiency of CAR lentivirus to patient T-cells was increased. We have determined that BX-795 increased CAR expression in T-cells 2- to 3-fold. However, transduction optimization among other transduction reagents/methods including the use of protamine sulfate, Vectofusin-1, and spinoculation along with BX-795 is yet to be studied to determine how much CAR-LV is reduced during CAR-T cell manufacturing. This has the prospect of facilitating more affordable CAR-T cell manufacturing in the future.

## Conclusion

In summary, the results of in vitro and in vivo efficacy and safety analyses in this study  demonstrated that ISIKOK-19 CAR-T cell production is robust, reproducible, and affordable for academic institutions. The findings will allow the introduction of the first clinical trial of ISIKOK-19 CAR-T cell treatment in relapsed/refractory ALL and NHL patients in Turkey.

## Figures and Tables

**Table 1:**
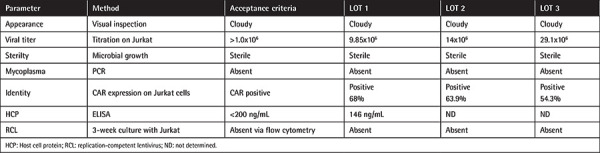
Quality control (sterility and efficacy) analysis of CAR lentivirus production lots (n=3).

**Table 2:**
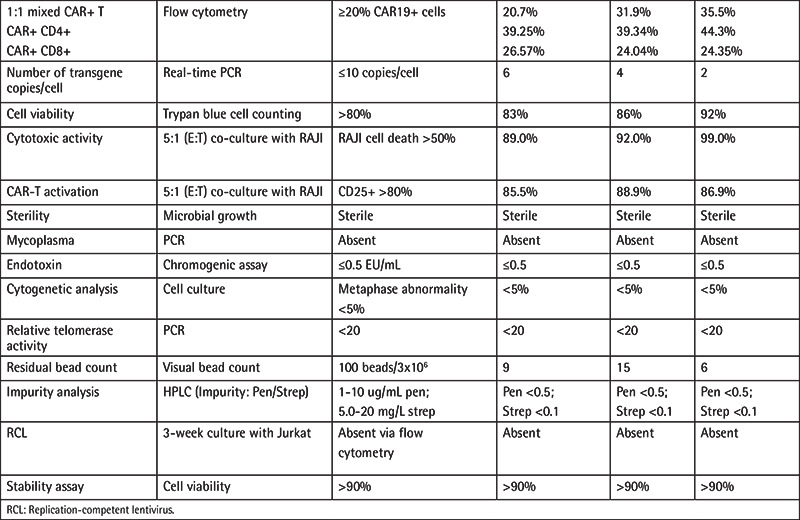
Quality control (sterility and efficacy) analysis of CAR-T cell production lots (n=3) from 3 healthy donors.
